# Biology of Superpowers: A Curriculum Activity for Teaching Adaptation, Trade-offs, and Organismal Diversity

**DOI:** 10.1093/iob/obag023

**Published:** 2026-05-18

**Authors:** A Eden

**Affiliations:** Biology Department, Salem State University, Salem, MA 01970, USA

## Abstract

Engaging students in integrative and organismal biology often requires instructional approaches that balance disciplinary rigor with accessibility and curiosity. Extraordinary traits in real organisms, such as regeneration in axolotls, echolocation in bats, or cryptobiosis in tardigrades, offer compelling entry points for exploring adaptation, evolutionary trade-offs, and the diversity of life. This article describes a curriculum activity, *Biology of Superpowers*, designed to leverage these traits to promote engagement, reinforce evolutionary concepts, and develop science communication skills in courses spanning introductory and upper-level biology. In this activity, students select a biological “superpower,” investigate its functional mechanisms and adaptive significance, and communicate their findings in a creative, public-facing format. An optional extension invites students to connect their chosen trait to superheroes or fictional characters, prompting critical evaluation of scientific accuracy and common misconceptions in popular media. The activity has been implemented in two distinct settings: an introductory non-majors *Diversity of Life* course and an upper-level *Evolution* course for biology majors. These dual contexts illustrate the adaptability of the assignment across levels. In the non-majors course, students emphasized accessible explanations and broad connections to survival and reproduction for general audiences, whereas majors more often incorporated mechanistic detail, comparative thinking, and consideration of constraints and trade-offs. Assessment is guided by revised rubrics that emphasize biological explanation, evolutionary reasoning appropriate to course level, clarity of communication, and use of evidence. Student artifacts are presented as illustrative examples of engagement with the activity rather than as formal evidence of learning gains. To strengthen implementation, the revised instructor and student materials include explicit prompts related to source evaluation, image attribution, appropriate AI use, and avoidance of common evolutionary misconceptions such as teleological reasoning or framing evolution as only about survival. Blank organizer templates are also provided to support scaffolding across course contexts. Together, these materials position *Biology of Superpowers* as a flexible curriculum model for teaching integrative and organismal biology by pairing curiosity-driven content with evolutionary thinking, evidence use, and science communication.

## Resumen en español

Involucrar a los estudiantes en la biología integrativa y organismal a menudo requiere enfoques pedagógicos que equilibren el rigor disciplinario con la accesibilidad y la curiosidad. Los rasgos extraordinarios en organismos reales, como la regeneración en ajolotes, la ecolocalización en murciélagos o la criptobiosis en tardígrados, ofrecen puntos de entrada atractivos para explorar la adaptación, los compromisos evolutivos y la diversidad de la vida. Este artículo describe una actividad curricular, *Biology of Superpowers*, diseñada para aprovechar estos rasgos con el fin de promover la participación estudiantil, reforzar conceptos evolutivos y desarrollar habilidades de comunicación científica en cursos de biología introductorios y avanzados. En esta actividad, los estudiantes seleccionan un “superpoder” biológico, investigan sus mecanismos funcionales y su significado adaptativo, y comunican sus hallazgos en un formato creativo dirigido a públicos no especializados. Una extensión opcional invita a los estudiantes a conectar el rasgo elegido con superhéroes o personajes ficticios, lo que promueve la evaluación crítica de la precisión científica y de conceptos erróneos comunes en los medios populares.

La actividad se ha implementado en dos contextos distintos: un curso introductorio de *Diversity of Life* para estudiantes no especializados en biología y un curso avanzado de *Evolution* para estudiantes de biología. Estos dos contextos ilustran la adaptabilidad de la actividad a distintos niveles. En el curso para estudiantes no especializados, los estudiantes enfatizaron explicaciones accesibles y conexiones amplias con la supervivencia y la reproducción para audiencias generales, mientras que los estudiantes de biología incorporaron con mayor frecuencia detalles mecanísticos, pensamiento comparativo y consideración de restricciones y compromisos evolutivos. La evaluación se guía por rúbricas revisadas que enfatizan la explicación biológica, el razonamiento evolutivo apropiado para el nivel del curso, la claridad de la comunicación y el uso de evidencia. Los productos estudiantiles se presentan como ejemplos ilustrativos de participación con la actividad, no como evidencia formal de ganancias de aprendizaje. Para fortalecer la implementación, los materiales revisados para instructores y estudiantes incluyen indicaciones explícitas relacionadas con la

evaluación de fuentes, la atribución de imágenes, el uso apropiado de IA y la evitación de conceptos evolutivos erróneos comunes, como el razonamiento teleológico o la idea de que la evolución se trata únicamente de supervivencia. También se proporcionan plantillas organizadoras en blanco para apoyar el andamiaje en distintos contextos de curso. En conjunto, estos materiales posicionan *Biology of Superpowers* como un modelo curricular flexible para enseñar biología integrativa y organismal al combinar contenido impulsado por la curiosidad con pensamiento evolutivo, uso de evidencia y comunicación científica.

## Introduction

Teaching integrative, organismal, and comparative biology offers rich opportunities for students to connect structure and function to ecology and evolution, yet these topics can feel abstract, especially when students encounter them primarily through lecture or decontextualized examples. In introductory courses for non-majors, learners may struggle to see how organismal biology relates to their lives or to contemporary issues, whereas majors may default to memorizing interesting details about organisms without consistently connecting traits to mechanism, adaptation, and constraint (Knight and Smith 2010). Prior work suggests that increasing students’ perceived relevance and providing opportunities to actively process ideas can support engagement and learning, particularly when students are asked to explain concepts in their own words and apply them in novel contexts (Brewer and Smith 2011; Freeman et al. 2014; Hymers and Newton 2019).

One approach to addressing these challenges is to harness the inherent “wonder” of biological diversity as an entry point into integrative thinking (Chodkowski et al. 2022). Extraordinary traits, such as regeneration, silent flight, extreme sensory abilities, bioluminescence, and physiological resilience, are immediately compelling to many students and provide concrete case studies for core organismal and evolutionary ideas. These traits naturally invite questions about how an ability works (mechanism), and how it may affect reproductive success and, when relevant, survival despite costs or constraints (trade-offs). Framed intentionally, “superpowers in nature” can support students in moving beyond fascination toward explanation, including comparative patterns such as convergence and divergence across taxa (Pryor 2008; Myer et al. 2024).

In parallel, there is growing recognition that biology courses can build students’ capacity to communicate science clearly and critically to audiences beyond the classroom (Brownell et al. 2013; McKinnon and Vos 2015). Public-facing communication products encourage students to organize ideas, make accurate claims with appropriate scope, and translate specialized language into accessible explanations. Popular media comparisons can further support this process by giving students a familiar reference point, while also creating an

authentic context for identifying scientific inaccuracies and misconceptions in how biology is portrayed (Pryor 2008; Eden 2025). Because students often encounter organismal traits through media, these comparisons can also provide a structured way to practice “myth-busting” and evidence-based reasoning (Ferguson et al. 2022).

At the same time, curiosity-driven activities such as these require careful scaffolding to avoid reinforcing common misunderstandings (Stern et al. 2018; Branch 2023). Students may default to unreliable sources, omit evidence attribution, rely on teleological language, or frame evolution primarily in terms of survival rather than differential reproductive success (Hammann and Nehm 2020). In contemporary classrooms, instructors may also need to help students use generative AI tools in limited and transparent ways that support, rather than replace, biological reasoning and communication. For these reasons, the revised version of this activity presented below and in supplemental materials includes explicit prompts related to source evaluation, image attribution, appropriate AI use, and avoidance of evolutionary misconceptions alongside blank planning organizers that support deeper analysis across course levels.

This article describes a curriculum activity, *Biology of Superpowers*, designed to leverage extraordinary traits as a flexible framework for teaching adaptation, diversity, and integrative organismal biology concepts while strengthening students’ science communication. In the activity, students select a biological “superpower,” investigate its functional mechanisms and adaptive significance, and communicate their findings through a creative product (e.g., infographic/mini-poster, short slide presentation, or brief written profile). An optional extension invites students to connect the trait to a superhero or fictional character and to evaluate what is accurate, exaggerated, or biologically implausible in that depiction. Implementation in both a non-majors Diversity of Life course and a majors Evolution course illustrates how the framework can be adapted across levels while maintaining common goals. Revised instructor and student materials are provided to support transferability and to make the activity easier for adopters to implement in ways that emphasize evidence use, scientific accuracy, and evolutionary reasoning.

## Activity description

### Overview

*Biology of Superpowers* is a flexible curriculum activity that uses extraordinary traits in real organisms as entry points for teaching integrative and organismal biology through structure–function reasoning, evolutionary explanation, and public-facing science communication. Students select a biological “superpower” such as regeneration, echolocation, magnetoreception, camouflage, or bioluminescence, investigate its mechanistic basis and adaptive significance, and then communicate their findings in a format appropriate for a broad audience. Because the traits students select span multiple organismal subdisciplines, the activity provides an integrative throughline that can be used across introductory and upper-level course contexts.

This activity was implemented during the Fall 2025 semester in two distinct settings: an introductory non-majors *Diversity of Life* course and an upper-level *Evolution* course for biology majors. In both contexts, students completed the activity individually, although the framework could be adapted for paired or small-group work with minimal modification. In the non-majors course, the activity emphasizes accessible explanations and clear connections between traits, organismal success, and basic evolutionary ideas. In the majors course, expectations emphasize mechanistic detail, evolutionary reasoning, and consideration of trade-offs and constraints. Table 1 summarizes the two course contexts and highlights the different assessment emphases used in each setting.

**Table 1:** Course contexts and implementation summary for *Biology of Superpowers*

Course context	Level/audience	Delivery mode	Approx. enrollment	Sharing format	Assessment approach
**Diversity of Life**	Intro/non-majors	In-person	27	Poster gallery/small groups	Rubric emphasizing trait explanation, connection to survival and reproduction, evidence use, communication, and creativity
**Evolution**	Upper-level/majors	Zoom synchronous	31	Breakouts + whole-class share-out	Rubric emphasizing scientific accuracy, evolutionary reasoning, evidence use, communication, and creativity

### Learning objectives

After completing the *Biology of Superpowers* activity, students will be able to:

1. Describe an extraordinary trait in a focal organism and explain how it functions using an appropriate level of detail for the course context.
2. Connect trait mechanism to organismal success by explaining how the trait may increase reproductive success and, when relevant, survival in a particular ecological setting.
3. Apply evolutionary reasoning to interpret the trait as an adaptation, including constraints and trade-offs; in majors contexts, students additionally consider comparative patterns such as convergence, divergence, or trait variation across taxa.
4. Communicate organismal biology to a public audience using a clear, creative product that is scientifically accurate and accessible.
5. *(Optional extension)* Critically evaluate a pop culture depiction of a similar “superpower,” distinguishing scientific plausibility from exaggeration or misconception and articulating what the depiction gets “right” or “wrong.”

These objectives align with integrative and organismal biology by emphasizing structure–function relationships across taxa and the evolutionary and ecological contexts that shape trait diversity.

### Activity procedure

The activity begins with a brief instructor-led introduction (approximately 10–15 min) that frames “superpowers” as real biological traits shaped by variation, selection, constraint, and ecological context. The instructor provides several examples to model the kinds of explanations students are expected to develop, including how a trait works, what it enables an organism to do, and what limits or trade-offs may accompany it. This opening framing is especially important for reducing the risk that students will describe traits as if organisms evolved them “on purpose,” a misconception that arose during initial implementation and is now addressed directly in the revised prompts and organizers.

Students then choose a focal trait and organism from a suggested list or propose their own topic with instructor approval. To support topic development, Table 2 serves as an instructor-facing example of how traits can be mapped onto integrative and organismal subdisciplines. Additionally, students received course-specific assignment sheets and blank planning organizers that prompted them to generate their own analyses of mechanism, function, sources, and, when appropriate, comparative patterns and pop culture connections. Separate sample organizer templates for majors and non-majors are provided in Supplementary File S3.

**Table 2:** Example “superpowers” mapped to organismal domains and integrative/organismal biology themes

“Superpower” trait (example organism)	Mechanism/domain emphasis	Integrative and organismal biology alignment	Common survival/reproduction framing	Possible pop culture anchor (optional)
Silk production and web engineering (spiders)	Biomechanics; protein biochemistry; behavior	Comparative biomechanics; comparative physiology/biochemistry; invertebrate zoology; ecology and evolution	Prey capture, egg protection, dispersal, habitat use	Spider-Man
Regeneration (axolotl; starfish; planarians)	Developmental processes; tissue repair physiology	Evo-devo; vertebrate morphology; invertebrate zoology; ecology, and evolution	Recovery from injury, continued function, trade-offs in time/energy	Wolverine/Deadpool
Echolocation (bats)	Sensory biology; neuroethology; physiology	Sensory biology; neurobiology; neuroethology; ecology and evolution	Navigation, foraging, mate finding, niche use	Batman
Camouflage/color change (chameleon; cuttlefish)	Physiology; sensory integration; behavior	Comparative physiology; sensory biology; neuroethology; ecology and evolution	Predator avoidance, communication, thermo-regulation	“Chameleon” portrayals
Silent flight (owls)	Biomechanics; feather morphology; behavior	Comparative biomechanics; vertebrate morphology; animal behavior; ecology and evolution	Prey capture and/or improved sensory tracking	“Silent assassin” tropes
Magnetoreception (sea turtles; migratory birds)	Sensory biology; navigation; neurobiology	Sensory biology; neurobiology; ecology and evolution; comparative biology	Long-distance navigation, migration, reproductive return to breeding sites	“Compass sense” characters
Bioluminescence (fireflies; jellyfish; deep-sea fish)	Biochemistry; signaling; behavior	Comparative physiology/biochemistry; animal behavior; ecology and evolution	Mate attraction, prey luring, predator deterrence	Glow/energy powers
Chemical defense (bombardier beetles; poison dart frogs)	Physiology/biochemistry; behavior	Comparative physiology/biochemistry; animal behavior; ecology and evolution	Predator deterrence, defense of self or offspring	Poison/acid-based characters

After selecting a topic, students conduct research guided by prompts tailored to course level. In *Diversity of Life*, students focus on identifying the organism, explaining the trait in simple but accurate language, describing how it may help the organism survive and reproduce, comparing it to at least one other organism, and avoiding goal-directed evolutionary phrasing. In *Evolution*, students address a parallel but more demanding set of prompts focused on mechanism, evolutionary significance, trade-offs and constraints, and comparative analysis. Both versions now include dedicated guidance on source credibility, image attribution, and AI use.

To support evidence-based communication, students are asked to use credible sources and to reflect on why those sources are trustworthy. In the revised majors version, students must use at least three credible sources, including at least one peer-reviewed source, and must include references and image credits in the final product. The revised non-majors assignment similarly requires credible sources, a reference list, and image credits, though with a lower overall source threshold and simpler source examples appropriate to course level. These revisions were made following implementation based on mixed results in student deliverables regarding evidence use and attribution and to make the activity more transferable for instructors seeking to emphasize scientific sourcing as part of public-facing biology communication.

The revised materials also address generative AI directly. Both course versions state that AI may be used only in limited ways, such as brainstorming or organizing ideas, and require students who use AI to submit a brief statement explaining how the tool was used and how the accuracy of the information was verified with credible sources. This addition responds to the practical reality that such assignments can be vulnerable to AI outsourcing and provides adopters with a concrete way to preserve the assignment’s emphasis on reasoning, communication, and evidence-based explanation.

The activity concludes with peer sharing and class synthesis. In the non-majors course, students shared work through an in-class poster gallery and small-group conversations. In the majors course, students shared in Zoom breakout rooms followed by whole-class discussion. In both contexts, the synthesis discussion emphasized structure–function relationships, adaptation, trade-offs, organismal diversity, and the difference between biologically plausible explanations and fictional exaggeration.

### Products

**Fig. 1:**
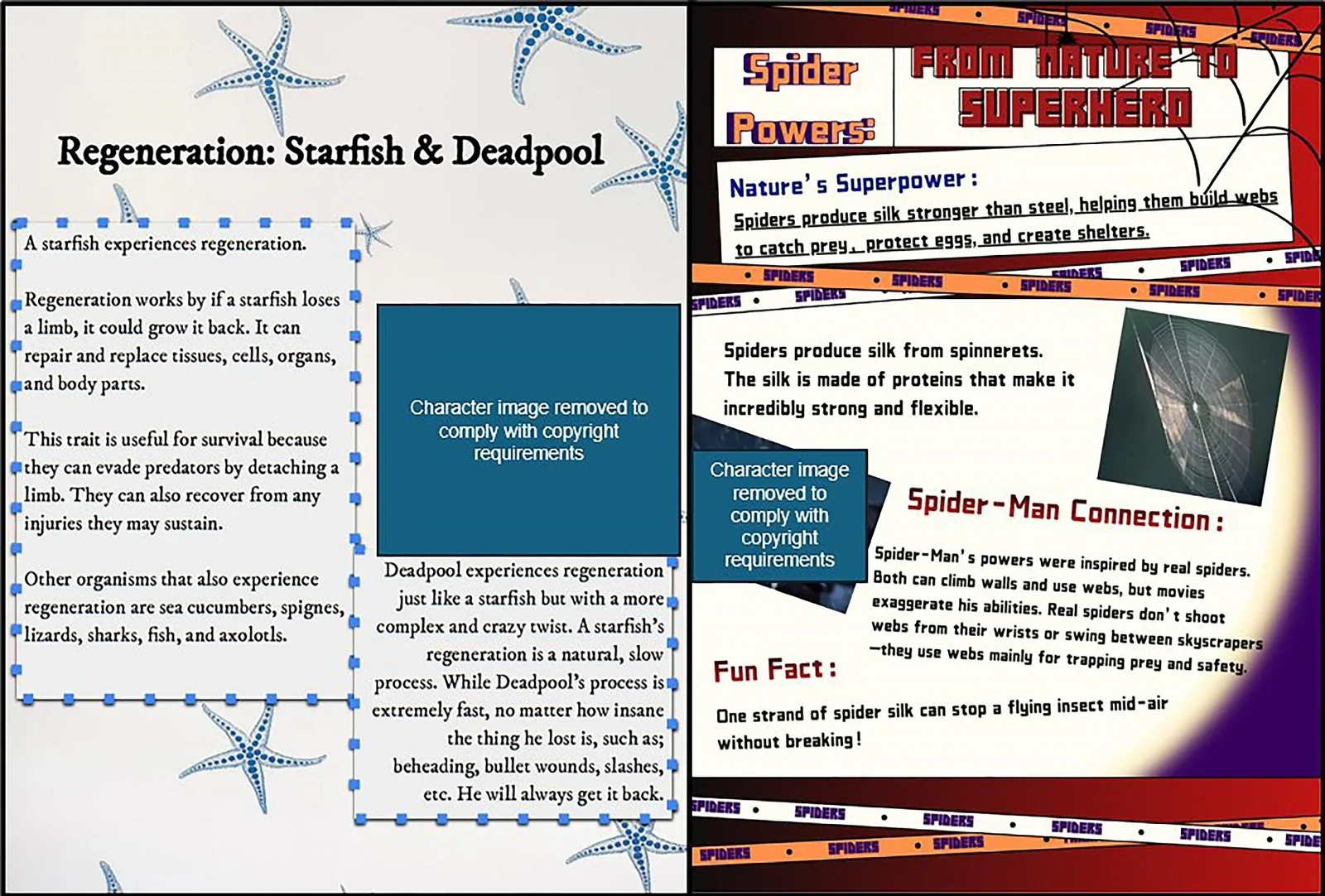
Example student products illustrating the optional pop culture extension, in which students compare a biological trait to a superhero/fictional depiction and identify plausible vs. exaggerated elements. Character images have been removed to comply with copyright requirements.

**Fig. 2:**
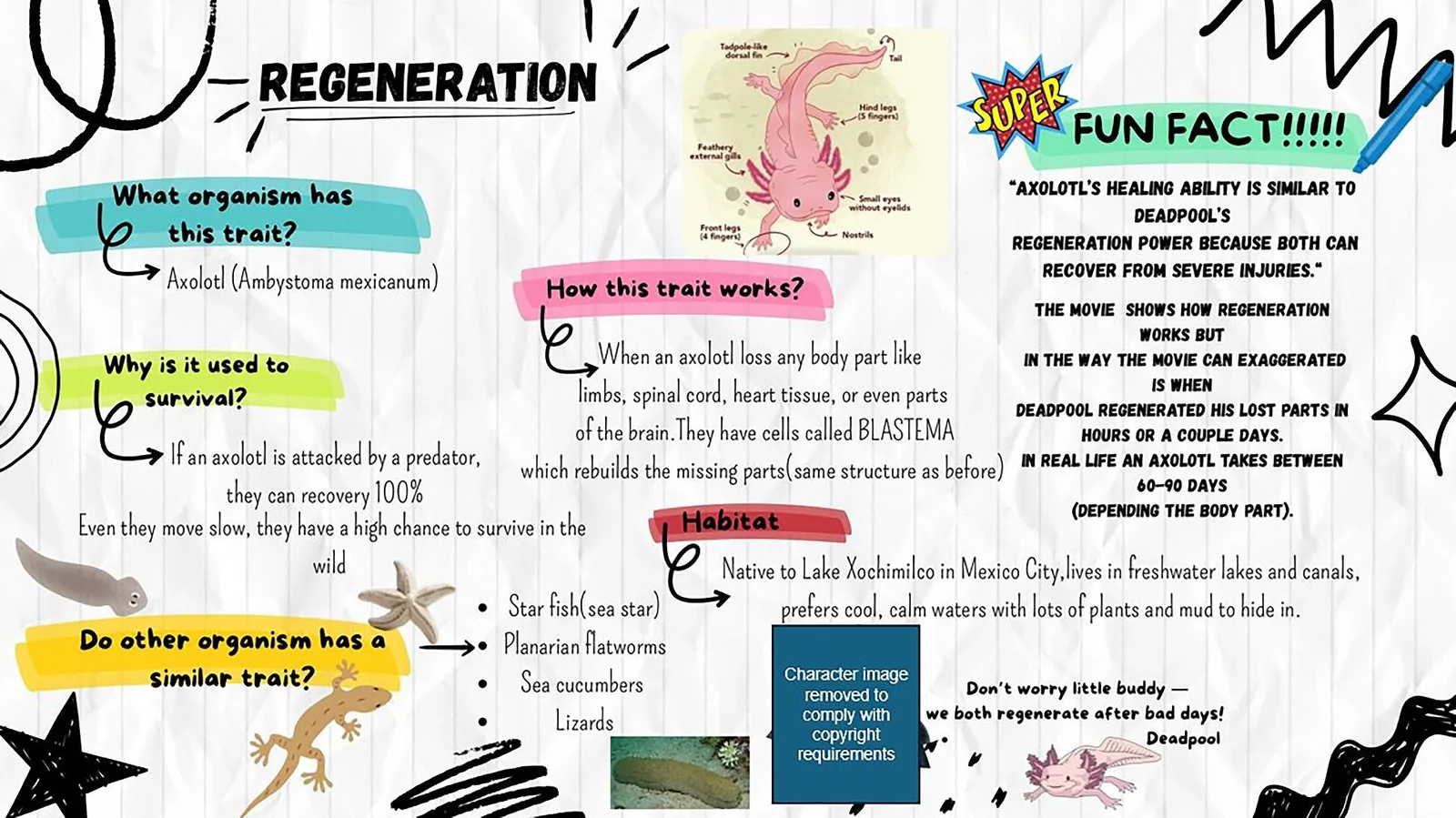
Representative non-majors submission (single-slide format) illustrating an accessible “how it works” explanation, how it may help an organism survive and reproduce, and optional pop culture comparison. Character images have been removed to comply with copyright requirements.

**Fig. 3:**
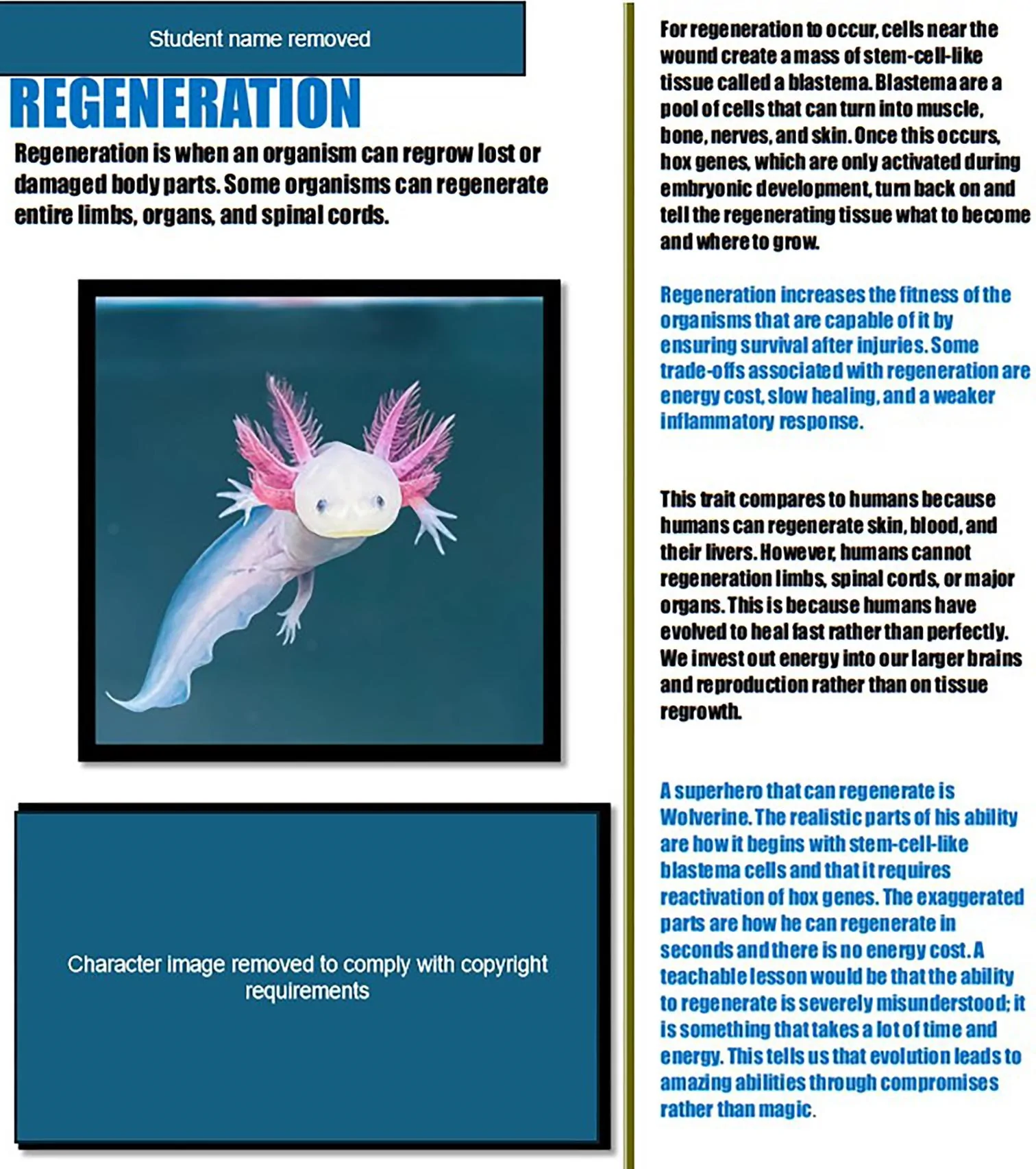
Representative majors-level submission from the initial implementation illustrating mechanistic detail, trade-offs, comparison to humans, and critique of fictional exaggeration in a regeneration-focused project. Character images have been removed to comply with copyright requirements.

Students select one of several formats to communicate their findings in a creative but scientifically accurate manner. In both course versions, options include an infographic/mini-poster, a short slide presentation delivered live or recorded, or a brief written profile in a science-communication style. This flexibility is pedagogically useful because it allows students to leverage different strengths while still meeting common conceptual goals, and it could also be understood through a Universal Design for Learning lens as offering multiple means of action and expression (CAST 2024). The optional pop culture extension remains an analytical add-on rather than the central task: students are first expected to explain the biology of the real organism, and only then compare it to a fictional counterpart. Example student products are shown in Figs. 1–3, including projects that incorporate the optional pop culture critique (Fig. 1), a representative non-majors submission (Fig. 2), and a majors exemplar that reflects mechanistic detail, trade-offs, and critique of fictional exaggeration (Fig. 3).

### Implementation notes

The activity is designed to be implemented in approximately 1 week, typically spanning one to two class sessions plus independent research and product development outside of class. It can be adapted for in-person or online instruction. In online settings, student products can be shared via a course discussion board, virtual gallery formats, or small-group breakout presentations. The activity could also be adapted for group work by asking students to collaborate on a shared organizer and divide responsibilities for research, source evaluation, and product design, although the implementation described here used individual submissions. This clarification may be helpful to instructors considering adaptations for larger classes or different course structures.

Scaffolding is particularly important for maintaining scientific accuracy while preserving creativity. The revised activity now provides multiple layers of support: course-specific prompts, revised rubrics, explicit guidance on evidence and attribution, AI-use expectations, and blank planning organizers. These supports are intended to reduce the likelihood that students will focus only on the “wow factor” of a trait without adequately explaining mechanism, organismal function, or evolutionary context. They also help address common student tendencies toward teleological language, survival-only framing, or overreliance on unvetted sources.

Finally, instructors planning to reproduce or publicly share student work should consider both copyright and attribution. Because students often choose pop culture examples that rely on copyrighted characters or media stills, the revised assignments explicitly require image credits and encourage attention to source quality. For publication purposes, text-based discussion of pop culture examples or use of openly licensed visuals may be preferable. These practical considerations make the activity easier to adopt responsibly in classrooms and easier to disseminate in scholarship of teaching and learning contexts.

## Assessment and observations

Student work in the *Biology of Superpowers* activity was assessed using course-specific rubrics designed to reflect the distinct goals of the two implementations. In the revised non-majors version, the rubric emphasizes trait explanation, connection to survival and reproduction, use of evidence and attribution, communication, and creativity. In the revised majors version, the rubric emphasizes scientific accuracy, evolutionary reasoning, use of evidence and attribution, clarity of communication, and creativity, with an optional pop culture extension in both contexts. Course-specific rubrics are provided in Supplementary Files S1 and S2, and Table 3 presents a general rubric framework for the activity.

**Table 3:** General rubric framework for *Biology of Superpowers*

Category	Excellent	Proficient	Developing	Points
**Accuracy of Explanation**	Trait is described correctly and clearly, with appropriate detail for the intended audience and course level.	Explanation is mostly correct, but some important details are underdeveloped or slightly unclear.	Explanation is inaccurate, vague, or too incomplete to understand clearly.	/20
**Connection to Reproduction, Survival, and Evolutionary Significance**	Clearly explains how the trait may help the organism reproduce, survive, protect offspring, or otherwise succeed in its environment; in advanced contexts, also addresses adaptation, trade-offs, constraints, or comparative patterns where appropriate.	Makes a basic connection to organismal success, but explanation lacks detail, depth, or consistency.	Connection to organismal success or evolutionary significance is weak, unclear, or missing.	/20
**Use of Credible Evidence and Attribution**	Uses the required number of credible sources, includes citations/references in a consistent format, and credits images appropriately. Evidence is used responsibly and accurately.	Uses sources and/or image credits, but some citations are incomplete, inconsistent, or weak/not clearly credible.	Sources are missing, unclear, not credible, or not appropriately connected to the project; citations and/or image credits are missing.	/10
**Communication and Clarity**	Product is organized, accessible, and appropriate for the intended audience while remaining scientifically accurate.	Product is mostly clear, though organization, readability, or accessibility could be improved.	Product is difficult to follow, too vague, or too technical for the intended audience.	/15
**Creativity and Engagement**	Format, visuals, examples, or framing enhance understanding and audience engagement.	Some creativity is present, but the product is more functional than engaging.	Minimal creativity or limited attention to audience engagement.	/15
**Pop Culture Extension (Optional)**	Thoughtful comparison clearly distinguishes biologically plausible elements from exaggeration or misconception.	Pop culture connection is present, but the analysis is limited or only partially developed.	Pop culture connection is weak, missing, or unclear.	+/5
**Total**				**/80 (+5)**

Because this manuscript is positioned as a curriculum article rather than a formal education research study, student artifacts are presented here as illustrative examples of how learners engaged with the activity, not as rigorous evidence of formal learning gains. Across both course contexts, students produced public-facing artifacts that generally reflected the central structure of the assignment: selecting an organismal trait, explaining how it works, and connecting that explanation to organismal success and communication for a broader audience. In the non-majors *Diversity of Life* course, student products most often emphasized accessible explanation, visual organization, and broad relevance to everyday understanding of biology. For example, the representative non-majors artifact shown in Fig. 2 organizes the explanation around questions such as what organism has the trait, how the trait works, and how it helps the organism, while also using the optional pop culture connection to distinguish between biological regeneration and fictional exaggeration. In future iterations, the revised non-majors prompts and rubric are intended to make links to reproduction and fitness more explicit than they were in some of the initial artifacts.

In the majors-level *Evolution* course, several submissions incorporated mechanistic detail and explicitly attended to plausibility, constraints, or comparative framing. The revised majors exemplar shown in Fig. 3, for instance, explains regeneration through blastema formation and Hox gene reactivation, identifies trade-offs such as energy cost and slow healing, compares regeneration in the focal organism to limited regeneration in humans, and critiques the unrealistic speed and cost-free nature of Wolverine’s fictional abilities. Importantly, this artifact does not demonstrate every dimension of the majors rubric, nor is it intended to do so. Rather, it is included as a more appropriate example of the kinds of mechanistic detail, comparison, and trade-off reasoning that the revised majors prompts and rubric now foreground.

The optional pop culture extension remained valuable across both contexts, but it functions best as an analytical add-on rather than the centerpiece of the assignment. In the initial implementation, pop culture comparisons often served as a natural entry point for discussing plausibility, timescale, and limits. In the revised materials, that role is made more explicit by asking students not only what fiction gets right or exaggerates, but also what misconceptions a fictional portrayal might reinforce. This shift helps align the activity with the goal of using pop culture as a scaffold for critical evaluation rather than as a substitute for biological explanation.

Taken together, the available artifacts suggest that the activity is effective at eliciting curiosity-driven engagement, public-facing explanation, and preliminary links between trait mechanism and organismal success. At the same time, the updated materials make clear that deeper learning about adaptation, fitness, trade-offs, and evolutionary misconception avoidance depends on the strength of the scaffolds provided. The materials are intended to make those expectations more visible and measurable for adopters. Future implementations could build on this foundation by pairing the rubric with brief pre/post prompts, structured reflection questions, or artifact coding focused on evidence use, mechanistic explanation, and the quality of evolutionary reasoning.

## Discussion

The *Biology of Superpowers* activity offers an adaptable curriculum model for teaching integrative and organismal biology by using extraordinary traits as accessible entry points into mechanism, function, and adaptation. Across both course contexts, the activity encouraged students to investigate how real traits work, why they may matter for organisms in their environments, and how those traits can be communicated to broader audiences in engaging formats. These outcomes are well aligned with the goals of organismal biology instruction, which often requires students to connect structure and function to ecology and evolution while also making sense of biological diversity in ways that feel relevant and memorable. In this respect, the activity’s central strength remains its ability to turn curiosity about unusual traits into a scaffold for biological explanation and communication.

A key strength of the activity is its flexibility across course levels. In the non-majors *Diversity of Life* course, the activity works well as an introduction to adaptation and trait diversity because it invites students to explain traits in accessible language and connect them to organismal success in familiar, concrete ways. In the majors-level *Evolution* course, the same basic framework can support deeper exploration of mechanism, trade-offs, comparative analysis, and the limits of fictional portrayals. The updated activity materials make this tiering explicit. The non-majors version now prompts students to connect traits to both survival and reproduction, rather than survival alone, while the majors version more directly asks students to address reproductive success, constraints, comparison across taxa, and avoidance of teleological language. These changes support the claim that the same curricular structure can be adapted meaningfully across audiences while preserving common goals.

The optional pop culture extension remains one of the most engaging elements of the activity. Reviewer feedback was helpful in revealing that pop culture comparisons can be pedagogically productive while also posing risks if they distract from mechanism or inadvertently reinforce misconceptions. In the revised activity, the fictional comparison is framed more deliberately as a means of evaluating biological plausibility, exaggeration, and misconception. This shift is reflected not only in the manuscript text but also in the revised prompts and rubrics, which now ask students to identify what fiction gets right, what it exaggerates, and what misunderstandings it might reinforce. This helps position pop culture as a scaffold for critical thinking rather than as a novelty element.

One of the most important revisions prompted by reviewer feedback is the stronger attention to evolutionary misconceptions, especially teleological language and survival-only explanations. Earlier versions of the activity left more room for students to describe traits as if organisms evolved them because they “needed” them or “developed” them for a purpose. The revised materials now address this directly by incorporating misconception checks into both course versions and by reframing prompts in terms of variation, selection, inheritance, and differential reproductive success. This change is particularly important because the activity asks students to create public-facing products, and such products can otherwise encourage oversimplification if the conceptual scaffolds are too loose. By naming these risks explicitly, the revised activity gives instructors clearer tools for helping students practice accurate, non-teleological evolutionary explanation.

The revised materials also address a practical concern that was absent from the original manuscript: how to implement this kind of assignment in the age of generative AI. Because a product-centered assignment can be vulnerable to outsourcing, the new versions make AI use transparent and bounded. Students may use AI in limited ways, such as brainstorming or organizing ideas, but they may not delegate the biological explanation or analysis to AI, and any AI use must be disclosed and verified against credible sources. This addition does not “solve” the challenge of AI in coursework, but it gives instructors a clear and adoptable starting point for preserving the assignment’s emphasis on explanation, critical thinking, and evidence-based communication.

Because this manuscript is presented as a curriculum article, not a formal education research study, a limitation is that outcomes are based on student artifacts and instructor observation rather than systematic learning-gain measures. Future iterations could add lightweight, instruction-friendly assessments that do not require extensive time or infrastructure, such as brief pre/post prompts on adaptation and trade-offs, a short misconception inventory tied to common fictional exaggerations, or structured reflection questions that probe how students’ understanding changed. Rubric-based coding of a sample of artifacts (e.g., presence of mechanism, explicit survival connection, mention of limits/trade-offs) could also provide a practical bridge between curriculum reporting and evidence of efficacy.

## Conclusion

Overall, *Biology of Superpowers* provides a transferable and engaging approach for teaching integrative and organismal biology in both introductory and upper-level contexts. By situating “superpowers” in real organisms, the activity invites students to connect mechanism to organismal success, appreciate the diversity of evolutionary solutions across taxa, and practice communicating biology in ways that resonate beyond the classroom without abandoning scientific rigor.

## Supplementary Material

obag023_Supplemental_Files

## Data Availability

No new research data were generated or analyzed in support of this article. The instructional materials associated with the curriculum activity are provided as supplementary data.
