# Supplementary material for: Biology of Superpowers: A Curriculum Activity for Teaching Adaptation, Trade-offs, and Organismal Diversity
Source: Integr Org Biol. 2026 May 18;8(1):obag023. doi: 10.1093/iob/obag023 (PMC13213587; doi:10.1093/iob/obag023)
Supplement: obag023_Supplemental_Files [file obag023_supplemental_files.zip › S1_Biology of Superpowers-majors_Evolution Course_REVISED.docx]

**Biology of Superpowers: Evolution in Action**

**Objective**

Many organisms have traits that seem like “superpowers,” from regeneration and echolocation to camouflage, magnetoreception, and survival in extreme environments. In this activity, you will investigate one of these traits, explain the biological mechanisms that make it possible, and analyze how the trait may have been shaped by natural selection. You will also have the option to connect your chosen trait to a superhero or pop culture character with a similar ability. By doing this, you will practice:

- Explaining biological traits through anatomy, physiology, behavior, genetics, or development.
- Using evolutionary reasoning to connect traits to reproductive success, survival, trade-offs, and constraints.
- Identifying comparative patterns such as convergence, divergence, or trait variation among related organisms.
- Communicating science clearly and responsibly to peers and broader audiences.

**Your Task**

Choose one extraordinary trait from a real organism.

Examples include:

- Regeneration (axolotls, planarians, starfish)
- Bioluminescence (fireflies, jellyfish, deep-sea fish)
- Extreme senses (echolocation in bats, infrared detection in snakes)
- Extreme strength or speed (leafcutter ants, mantis shrimp, peregrine falcons)
- Physiological extremes (antifreeze proteins in polar fish, tardigrade cryptobiosis)
- Defense superpowers (poison dart frogs, camouflage in cuttlefish)

**Research and Explain**

Your project must address the following questions:

**1. Mechanism**
What anatomical, physiological, behavioral, developmental, or genetic mechanisms make this trait possible?

**2. Evolutionary significance**
How might this trait increase reproductive success and, when relevant, survival? Be specific about what the organism gains in its environment.

**3. Trade-offs and constraints**
What costs, limitations, or constraints are associated with this trait? Why do not all organisms have it?

**4. Comparative analysis**
How does this trait compare to humans, close relatives, or distantly related organisms? If a similar trait appears in unrelated groups, could this reflect convergent evolution?

**5. Misconception check**
Avoid goal-directed or teleological language such as “the organism developed this trait in order to…” Instead, explain the trait in terms of variation, selection, inheritance, and differential reproductive success.

**Evidence and Sources**

Use at least **3 credible sources**, including:

- At least **1 peer-reviewed scientific source** or review article.
- No more than **1 general reference source** (i.e., Britannica) unless approved.

For each source, ask:

- Who wrote it?
- What expertise or institution is behind it?
- Is the claim supported by evidence?
- Is the source scientific, educational, or peer reviewed?

You must include:

- **References** section or final slide with your sources in a consistent format.
- **Image credits** for all images that are not your own.

Your references list may use **APA or MLA style**. Choose one style and use it consistently. For help formatting citations, you may use the [Purdue OWL APA Formatting and Style Guide](https://owl.purdue.edu/owl/research_and_citation/apa_style/apa_formatting_and_style_guide/index.html?) or [MLA Formatting and Style Guide](https://owl.purdue.edu/owl/research_and_citation/mla_style/mla_formatting_and_style_guide/index.html?).

**AI Use**

You may use AI tools for limited support such as brainstorming, outlining, or helping you rephrase your own ideas. You may **not** use AI to generate your full explanation, analysis, or comparison for you. If you use AI in any way, include a brief **AI Use Statement** at the end of your project that explains what tool you used, how you used it, and how you checked the accuracy of the information with credible sources. You are responsible for the accuracy of everything you submit.

**Deliverable**

Choose one format:

- Mini-poster / infographic aimed at a general audience.
- Short slide deck (3–5 slides) with recorded narration.
- Brief written science-communication profile (roughly 700–1000 words).

**Optional / Extra Credit: Pop Culture Connection**

Identify a superhero or fictional character with a similar trait. Compare science and fiction:

- What is biologically plausible?
- What is exaggerated or impossible?
- What misconceptions might the fictional version reinforce?

**Rubric**

| **Category** | **Excellent** | **Proficient** | **Developing** | **Points** |
| --- | --- | --- | --- | --- |
| **Scientific Accuracy** | Trait mechanisms are correct, detailed, and clearly explained using appropriate biological language. Explanations are accurate and well supported. | Explanation is mostly correct, but some details are underdeveloped or slightly unclear. | Explanation contains inaccuracies, major gaps, or unclear biological reasoning. | /20 |
| **Evolutionary Reasoning** | Clearly explains how the trait may affect **reproductive success** and, when relevant, survival. Thoughtfully addresses adaptation, constraints, trade-offs, and comparative patterns such as convergence/divergence where appropriate. | Addresses evolutionary significance and may mention trade-offs or comparison, but explanation lacks depth, specificity, or consistency. | Evolutionary explanation is minimal, vague, or inaccurate. Focuses only on survival or uses teleological reasoning without correction. | /20 |
| **Use of Credible Evidence and Attribution** | Uses the required number of credible sources, includes citations/references in a consistent format, and credits images appropriately. Evidence is used responsibly and accurately. | Uses sources and/or image credits, but some citations are incomplete, inconsistent, or some sources are weak/not clearly credible. | Sources are missing, unclear, not credible, or not appropriately connected to the project; citations and/or image credits are missing. | /10 |
| **Clarity of Communication** | Product is well organized, engaging, and accessible to non-experts while remaining scientifically accurate. | Product is generally clear, but organization, readability, or accessibility could be improved. | Product is difficult to follow, too vague, or too technical for the intended audience. | /15 |
| **Creativity and Engagement** | Format, visuals, examples, or framing strongly enhance understanding and audience engagement. | Some creativity is present, but the format is more functional than engaging. | Minimal creativity or little attention to audience engagement. | /15 |
| **Pop Culture Extension (Optional)** | Comparison to superhero/fictional portrayal is thoughtful and accurate, clearly distinguishing plausible biology from exaggeration or misconception. | Pop culture connection is present but analysis is limited or only partially accurate. | Pop culture connection is weak, missing, or does not meaningfully analyze science vs. fiction. | +/5 |
| **Total** | | | **/80 (+5)** | |
