# Supplementary material for: Biology of Superpowers: A Curriculum Activity for Teaching Adaptation, Trade-offs, and Organismal Diversity
Source: Integr Org Biol. 2026 May 18;8(1):obag023. doi: 10.1093/iob/obag023 (PMC13213587; doi:10.1093/iob/obag023)
Supplement: obag023_Supplemental_Files [file obag023_supplemental_files.zip › S2_Biology of Superpowers-Non-Majors_REVISED.docx]

**Biology of Superpowers: Evolution in Action**

**Objective**

The natural world is full of amazing traits that can seem like “superpowers.” In this project, you will choose one extraordinary trait from a real organism, explain how it works, and describe how it may help that organism survive, reproduce, or leave offspring in its environment. You may also connect your trait to a superhero or pop culture character with a similar power.

By completing this project, you will:

- Discover the diversity of adaptations across living things.
- Explain how traits work in understandable, accurate language.
- Connect traits to what helps organisms survive and reproduce (increase fitness).
- Practice communicating science to an audience outside the classroom.

**Your Task**

Choose one extraordinary trait from a real organism or suggest your own with instructor approval. Examples:

- Regeneration (starfish regrow arms, lizards regrow tails)
- Bioluminescence (fireflies, anglerfish)
- Super senses (bats using echolocation, bees seeing UV light)
- Super strength or speed (ants, peregrine falcons)
- Survival in extreme environments (tardigrades, camels, polar bears)
- Defense powers (poison dart frogs, bombardier beetles, camouflage in cuttlefish)

**Research and Explain**

Answer the following in your project:

**1. What organism has this trait?**

**2. How does the trait work?**
Explain it in simple, accurate language.

**3. How might this trait help the organism survive and reproduce?**
This could include helping it find food, avoid predators, attract mates, protect offspring, or live successfully in its environment.

**4. Do other organisms have a similar trait?**
Give at least one comparison.

**5. Misconception check**
Do not write as if organisms “needed” a trait and then evolved it on purpose. Instead, explain that traits become common over time when they help organisms leave more offspring.

**Sources and Images**

Use at least **2 credible sources**. Good options include:

- Museum, university, or government science websites.
- Textbooks or course materials.
- Peer-reviewed or science-based articles.

You must include:

- **References** list at the end.
- **Image credits** for any image you did not create yourself.

Your references list may use **APA or MLA style**. Choose one style and use it consistently. For help formatting citations, you may use the [Purdue OWL APA Formatting and Style Guide](https://owl.purdue.edu/owl/research_and_citation/apa_style/apa_formatting_and_style_guide/index.html?) or [MLA Formatting and Style Guide](https://owl.purdue.edu/owl/research_and_citation/mla_style/mla_formatting_and_style_guide/index.html?).

**AI Use**

You may use AI tools for limited support such as brainstorming, outlining, or helping you rephrase your own ideas. You may **not** use AI to generate your full explanation, analysis, or comparison for you. If you use AI in any way, include a brief **AI Use Statement** at the end of your project that explains what tool you used, how you used it, and how you checked the accuracy of the information with credible sources. You are responsible for the accuracy of everything you submit.

**Deliverable**

Choose one format:

- Poster or infographic.
- Short slide deck (3–5 slides) with recorded narration.
- Short written museum-style profile.

**Optional / Extra Credit: Pop Culture Connection**

Find a superhero or fictional character with a similar “superpower.”
Explain:

- What did the movie, show, or book get right?
- What did it exaggerate or get wrong?

**Rubric**

| **Category** | **Excellent** | **Proficient** | **Developing** | **Points** |
| --- | --- | --- | --- | --- |
| **Trait Explanation** | Clearly explains how the trait works in simple, accurate language appropriate for a general audience. | Explanation is mostly correct, but some details are vague or underdeveloped. | Explanation is confusing, inaccurate, or too incomplete to understand clearly. | /20 |
| **Connection to Survival and Reproduction** | Clearly explains how the trait may help the organism survive, reproduce, protect offspring, or succeed in its environment. | Makes a basic connection to survival or reproduction, but the explanation lacks detail or clarity. | Connection to survival/reproduction is weak, unclear, or missing. | /20 |
| **Use of Credible Evidence and Attribution** | Uses the required number of credible sources, includes citations/references in a consistent format, and credits images appropriately. Evidence is used responsibly and accurately. | Uses sources and/or image credits, but some citations are incomplete, inconsistent, or some sources are weak/not clearly credible. | Sources are missing, unclear, not credible, or not appropriately connected to the project; citations and/or image credits are missing. | /10 |
| **Communication and Clarity** | Product is organized, easy to follow, and accessible for non-scientists. | Product is mostly clear, though organization or accessibility could be improved. | Product is difficult to follow, too vague, or too technical. | /15 |
| **Creativity and Engagement** | Product is visually or rhetorically engaging and helps the audience stay interested. | Some creativity is present, but the product is mostly functional. | Minimal creativity or limited effort to engage the audience. | /15 |
| **Pop Culture Extension (Optional)** | Thoughtful comparison identifies what is accurate and what is exaggerated in the fictional example. | Pop culture connection is present but analysis is limited. | Pop culture connection is weak, missing, or unclear. | +/5 |
| **Total** | | | **/80 (+5)** | |
