# Supplementary material for: Biology of Superpowers: A Curriculum Activity for Teaching Adaptation, Trade-offs, and Organismal Diversity
Source: Integr Org Biol. 2026 May 18;8(1):obag023. doi: 10.1093/iob/obag023 (PMC13213587; doi:10.1093/iob/obag023)
Supplement: obag023_Supplemental_Files [file obag023_supplemental_files.zip › S3_Organizer Templates.docx]

**Biology of Superpowers Organizer Templates**

*Student planning templates for majors and non-majors implementations*

**Organizer 1. Majors (Evolution Course)**

| **Getting to know your organism** | |
| --- | --- |
| **Scientific name** |  |
| **Common name** |  |
| **Organism group**  *(mammal, insect, plant, etc.)* |  |
| **Learning about the “superpower”** | |
| **Mechanism**  *(What anatomical, physiological, behavioral, developmental, or genetic mechanisms make this trait possible?)* |  |
| **Evolutionary significance**  *(How might this trait increase reproductive success and, when relevant, survival? Be specific about what this trait helps the organism do in its environment.)* |  |
| **Trade-offs and constraints**  *(What costs, limitations, or constraints might come with this trait? Why do not all organisms have it?)* |  |
| **Comparative analysis** | |
| **How does this trait compare to humans?** |  |
| **How does this trait compare to the organism’s close relatives?** |  |
| **How does this trait compare to distantly related organisms with a similar trait?** |  |
| **Could this be an example of convergent evolution? Why or why not?** |  |
| **Evidence and sources** | |
| **Source 1** |  |
| **Source 2** |  |
| **Source 3** |  |
| **What source is peer-reviewed?** |  |
| **What makes your sources credible?** |  |
| **Deliverable planning** | |
| **What format will your deliverable be?** | ☐ Infographic / mini-poster  ☐ Slide deck with narration  ☐ Written science-communication profile |
| **Who is your audience?** |  |
| **What 3 points do you most want your audience to understand?** |  |
| **Optional pop culture connection** | |
| **What fictional character or pop culture example relates to this trait?** |  |
| **What does fiction get right?** |  |
| **What does fiction exaggerate or get wrong?** |  |
| **AI use statement** | |
| **If you used AI for brainstorming, outlining, or revising, explain how you used it and how you verified accuracy with reliable sources.** |  |

**Organizer 2. Non-majors course (Slightly modified)**

| **Getting to know your organism** | |
| --- | --- |
| **Scientific name** |  |
| **Common name** |  |
| **Organism group**  *(mammal, insect, plant, etc.)* |  |
| **Where is the organism found?** |  |
| **Learning about the “superpower”** | |
| **How does the trait work?**  *(Explain the trait in simple, accurate language.)* |  |
| **How might this trait help the organism survive and reproduce?**  *(Think about things like finding food, escaping predators, attracting mates, protecting offspring, or living successfully in its environment.)* |  |
| **Comparing to other organisms** | |
| **List at least one other organism with a similar trait** |  |
| **How is it similar or different?** |  |
| **Evidence and sources** | |
| **Source 1** |  |
| **Source 2** |  |
| **Source 3** |  |
| **Why do you think these are trustworthy?** |  |
| **Deliverable planning** | |
| What format will your deliverable be? | ☐ Infographic / mini-poster  ☐ Slide deck with narration  ☐ Written science-communication profile |
| **Who is your audience?** |  |
| **What 3 points do you most want your audience to understand?** |  |
| **Optional pop culture connection** | |
| **What fictional character or pop culture example relates to this trait?** |  |
| **What does fiction get right?** |  |
| **What does fiction exaggerate or get wrong?** |  |
| **AI use statement** | |
| **If you used AI for brainstorming, outlining, or revising, explain how you used it and how you verified accuracy with reliable sources.** |  |
